# A Fungal Defensin Targets the SARS−CoV−2 Spike Receptor−Binding Domain

**DOI:** 10.3390/jof7070553

**Published:** 2021-07-12

**Authors:** Bin Gao, Shunyi Zhu

**Affiliations:** Group of Peptide Biology and Evolution, State Key Laboratory of Integrated Management of Pest Insects and Rodents, Institute of Zoology, Chinese Academy of Sciences, 1 Beichen West Road, Chaoyang District, Beijing 100101, China; gaob@ioz.ac.cn

**Keywords:** micasin, coronavirus, SARS−CoV−2, dermatophytic fungus, antiviral drug

## Abstract

Coronavirus Disease 2019 (COVID−19) elicited by the Severe Acute Respiratory Syndrome Coronavirus 2 (SARS−CoV−2) is calling for novel targeted drugs. Since the viral entry into host cells depends on specific interactions between the receptor−binding domain (RBD) of the viral Spike protein and the membrane−bound monocarboxypeptidase angiotensin converting enzyme 2 (ACE2), the development of high affinity RBD binders to compete with human ACE2 represents a promising strategy for the design of therapeutics to prevent viral entry. Here, we report the discovery of such a binder and its improvement via a combination of computational and experimental approaches. The binder micasin, a known fungal defensin from the dermatophytic fungus *Microsporum canis* with antibacterial activity, can dock to the crevice formed by the receptor−binding motif (RBM) of RBD via an extensive shape complementarity interface (855.9 Å2 in area) with numerous hydrophobic and hydrogen−bonding interactions. Using microscale thermophoresis (MST) technique, we confirmed that micasin and its C−terminal γ−core derivative with multiple predicted interacting residues exhibited a low micromolar affinity to RBD. Expanding the interface area of micasin through a single point mutation to 970.5 Å2 accompanying an enhanced hydrogen bond network significantly improved its binding affinity by six−fold. Our work highlights the naturally occurring fungal defensins as an emerging resource that may be suitable for the development into antiviral agents for COVID−19.

## 1. Introduction

Severe acute respiratory syndrome corona virus 2 (SARS−CoV−2) is the pathogen of Coronavirus Disease 2019 (COVID−19), a recently emerged worldwide pandemic that is causing a global health crisis. Although the urgent usage of vaccines has achieved some initial success in controlling the disease spread, targeted medicines as a therapeutic means are still indispensable [1]. Unfortunately, no SARS−CoV−2 targeted medicine has been successfully applied clinically for the treatment of COVID−19 at the current stage. Nevertheless, numerous studies over the past year have made encouraging progress in developing targeted drug leads for COVID−19. For example, a structure−based design has led to the discovery of two promising inhibitors against the main protease (M^pro^) of SARS−CoV−2, a key enzyme mediating viral replication and transcription [2,3]. In addition to the M^pro^, SARS−CoV−2 Spike protein is becoming an even more attractive target for exploiting targeted drugs. This trimeric protein protrudes from the double−layered lipid envelope of the SARS−CoV−2 virion and is responsible for viral entry into the host cell through the receptor−binding domain (RBD) to recognize and bind the membrane−bound monocarboxypeptidase angiotensin converting enzyme 2 (ACE2) [4]. Targeting the protein−protein interactions (PPIs) via competitively binding the RBD has been proved to be efficient in blocking the initial stage of virus entry. Such binders in development include engineered ACE2 decoys, monoclonal antibodies, small compounds or peptides, and vaccine−elicited antibodies or convalescent serum [5].

Compared with the much larger decoys and antibodies, peptides have their own advantages as therapeutic agents due to their lower cost, minimal immunogenicity and potentially lower toxicity [6,7]. For the development of RBD−targeted peptides, one strategy is to replicate the viral binding region on ACE2, essentially based on the interacting residue−rich helix 1 [8]. De novo design of peptides to competitively bind RBD with ACE2 even exhibited more potency [9]. The other strategy is to screen antiviral candidates from the naturally occurring bioactive peptides. In this aspect, antimicrobial peptides (AMPs) are attracting increasing attention with regard to their possibility of blocking cellular surface receptors and/or disruption of virus cell membrane at the stage of virus entry [9,10,11,12,13,14]. As the effectors of the innate immune system of multicellular organisms, AMPs establish the first−line defense towards microbial infection and are being exploited as alternatives of conventional anti−infective agents to combat bacterial and fungal infections [15]. The cathelicidin LL37 and defensins (both α− and β−types) are two classes of major naturally occurring AMPs in humans that contribute to both innate and adaptive antimicrobial immunity [16]. Recent studies have identified their potential roles in resistance to SARS−CoV−2 infection. For example, human β−defensin 2 (hBD−2), an epithelial cell−derived AMP, binds to RBD with a K_D_ of ~300 nM [17]. Human intestinal α−defensin 5 (HD−5) and LL37 can simultaneously attach both ACE2 and the Spike protein [18,19,20]. A defensin−like peptide P9R exhibited potent antiviral activity against multiple viruses including SARS−CoV−2. The antiviral activity of P9R depends on the direct binding to viruses and the inhibition of virus−host endosomal acidification [21].

In addition to these human−sourced AMPs, defensins with the cysteine−stabilized α−helical and β−sheet (CSαβ) motif represent a much larger family of AMPs conserved across fungi, invertebrates and plants [22,23,24,25] but they have not been tested for antiviral activity against SARS−CoV−2. Compared with the membrane−disruptive linear AMPs, this class of peptides show higher stability and lower mammalian toxicity due to the presence of multiple disulfide bridges and their specific interactions with certain microbial targets [26,27]. Micasin is such a peptide characterized from the dermatophytic fungus *Microsporum canis* [24]. In this work, we first studied the potential interactions of micasin and RBD through molecular docking. The results showed that this peptide could dock on the RBD receptor−binding motif to form a stable complex via extensive shape complementarity. Subsequently, we experimentally validated the interactions with microscale thermophoresis (MST). Further single−point mutation guided by the structural complex information to expand the interface results in a mutant with six−fold higher binding affinity.

## 2. Materials and Methods

### 2.1. Construction and Analysis of the Complex between Micasin and RBD

The HEX program (version 8.0.0) (http://hex.loria.fr/, accessed on 10 March 2020) that uses a fast Fourier transform (FFT)−based rigid−body docking algorithm to perform protein−protein docking [28] was used to establish an initial complex between micasin (pdb entry 2LR5) and RBD (pdb entry 6LZG) [29]. The first docking pose was chosen for energy minimization using the force filed of AMBER 14 implemented in Molecular Operating Environment (MOE) 2019.0102 (https://www.chemcomp.com/index.htm, accessed on 10 March 2020). The interface area of a complex was calculated as the difference in total accessible surface areas of isolated and interfacing structures divided with PDBePISA (https://www.ebi.ac.uk/msd-srv/prot_int/pistart.html, accessed on 18 April 2021). Hydrogen bonds and hydrophobic interactions in the energically minimized complex were recognized by LigPlot+ [30] where hydrogen−bond calculation parameters are 2.70 (maximum: H−A distance) to 3.35 (maximum D−A distance) (here, H = hydrogen; A = acceptor; D = donor). Structure figures were made using MolMol 2k.2 [31] or PyMOL 2.4 (https://pymol.org/2/, accessed on 10 March 2020).

### 2.2. Molecular Dynamics (MD) Simulations

A 40−ns of MD simulations for E8R in its unbound and bound states to RBD were performed with the GROMACS 2020.1 software package (http://www.gromacs.org/, accessed on 25 March 2020) using the OPLS (Optimized Potential for Liquid Simulations) −AA/L all−atom force field (2001 aminoacid dihedrals) and TIP3P model for explicit water. Solvent shell thickness was 1.5 nm in a cubic box and the total charge of the simulated systems were neutralized by adding sodium or chloride ions. Simulations were performed under periodic boundary conditions and energy minimization by the steepest descent minimization algorithm with the maximum force <1000.0 kJ/mol/nm. The system was subsequently equilibrated with a two−step protocol that involved a 100 ps number of particles, volume, and temperature (NVT) and a 100 ps number of particles, pressure, and temperature (NPT), in which all protein atoms were restrained to their initial position. Following these two equilibration stages, a 30 ns trajectory was simulated (i.e., production MD) with a time step of 2 fs. The modified Berendsen thermostat method was used to maintain the temperature at 300 K and pressure coupling was performed with the Parrinello−Rahman algorithm for 1 bar. The Particle Mesh Ewald (PME) algorithm was used to treat long−range electrostatic interactions and the Linear Constraint Solver (LINCS) algorithm to constrain all bonds. Short−range electrostatic and short−range van der Waals cutoffs were both 1 nm. The root mean squared deviation (RMSD) for measuring the difference of simulated structures to the structure present in the minimized, equilibrated system were calculated with the gmx rms command of Gromacs.

### 2.3. Construction of Computational Model of (BH)−1

**Figure 1:**
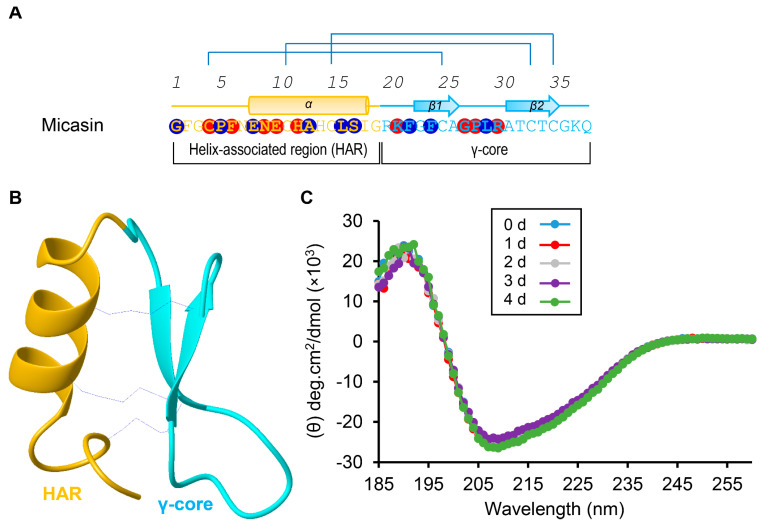
Micasin is a thermally stable CSαβ−type defensin. (**A**) The primary and secondary structures extracted from the structural coordinates of micasin (pdb entry 2LR5). Three disulfide bridges are indicated by lines in blue; α and β represent α−helix and β−strand, respectively. In panels A and B, two subdomains as the N−terminal helix−associated region (HAR) and the C−terminal γ−core region are shown in different colors and circled residues shaded in red and blue represent their contacts with RBD by hydrogen−bonding and hydrophobic interactions, respectively (see Figure 2). (**B**) The ribbon model of micasin resolved from NMR (24). The color scheme is the same as that of panel A. (**C**) Far−ultraviolet CD spectra of micasin after treated 0–4 days at 50 °C.

The structure of (BH)−1 was firstly built from micasin via deleting its N−terminal HAR (see Figure 1A) by PyMOL 2.4 and then energy minimization by MOE2019.0102 with the force field AMBER 14. The mutation from the Gly to Cys (Figure 1A and Figure 4A) had a Δ_stability_ of −3.24 based on the calculation of Disulfide Scan of MOE2019.0102. The mutation-mediated disulfide bridge connection was performed by Swiss-PdbViewer 4.1 (https://spdbv.vital-it.ch/, accessed on 10 March 2020) and further energy minimization carried out by MOE2019.0102.

### 2.4. Oxidative Refolding of Chemically Synthesized Micasin Mutants

Micasin and mutants (E8K, E8R, E8W and (BH)−1) were chemically synthesized in a reduced form by ChinaPeptides Co., Ltd. (Shanghai, China) with purity >95%. For oxidative refolding, a synthetic peptide was first dissolved in water in a concentration of 2 mg/mL and then 100 mM Tris−HCl (pH 8.0–8.5) was used to dilute the peptide solution to a final concentration of 0.1–0.2 mg/mL. The solution was incubated at 25 °C 48 h and the oxidized product was then purified by reverse−phase high performance liquid chromatographic (RP−HPLC). The collected peak was lyophilized by Thermo Scientific SAVANT SPD1010 SpeedVac Concentrator (Thermo Fisher Scientific, Waltham, USA). The purity and molecular weight was identified by matrix−assisted laser desorption/ionization time of flight mass spectrometry (MALDI−TOF) using an Ultraflextreme^TM^ instrument (Bruker Daltonics, Bremen, Germany) in the positive−ion mode and α−cyano−4−hydroxycinnamic acid (CHCA) as a liquid matrix.

### 2.5. Renaturation of RBD from E. coli Inclusion Body

The codon−optimized gene coding for SARS−CoV−2 Spike RBD (residues N^334^−P^527^) (Wuhan seafood market pneumonia virus isolate Wuhan−Hu−1, GenBank: MN908947.3) (herein abbreviated as RBD) was synthesized using DNA microarray synthesis technology from the Tsingke company (Beijing, China) and ligated into pET−28a by *Nco* I and *Sal* I restriction enzyme sites. The recombinant plasmid pET−28a−RBD was transformed into *E. coli* BL21(DE3) for auto−induction to accumulate inclusion bodies (IBs) under the direction of the T7 promoter. The IBs were then renatured by the previously described method with some modifications [32]. In brief, the auto−induction overnight culture was centrifuged and the pellet was resuspended in the resuspension buffer (RB, 100 mM Tris−HCl, pH 8.5 and 100 mM NaCl) followed by ultrasonic disruption to prepare bacterial lysates, from which crude IBs were collected by centrifugation. The IBs were washed twice with 2% Triton X−100 in the RB buffer and then solubilized by 6 M guanidine−HCl in 0.1 M Tris−HCl (pH 8.5), 1 mM EDTA and 30 mM β−mercaptoethanol for 1 h at room temperature. Renaturation was initiated by a 20 times of dilution of the solubilized IBs into the renaturation buffer containing 0.2 M ammonium acetate (pH 8.8), 10% glycerol and 1 M guanidine−HCl. Following incubation at 16 °C overnight, the precipitates in the renaturation solution were removed by filtering with the Whatman filter papers. Subsequently, the protein solution was ultrafiltered to exchange buffer to 0.2 M ammonium acetate (pH 8.8) with a 3 kD MWCO centrifugal device (Millipore, Bedford, MA, USA). The sample was analyzed by sodium dodecyl sulfate–polyacrylamide gel electrophoresis (SDS−PAGE). Further purification was carried out by size−exclusion chromatography (SEC) with a Superdex^TM^ 75 Increase 10/300 GL column on an AKTA Pure 25 system (GE Healthcare Life Sciences, Pittsburgh, PA, USA) with 50 mM Sodium Phosphate, pH 7.0, 150 mM NaCl as the running buffer with a flow rate of 0.3 mL min^−1^. Peak fractions were pooled and protein concentration was determined by measuring the absorbance of the protein solution at 280 nm with a UV−VIS Spectrophotometer (NanoDrop2000). The sample was stored at −80 °C for use. The feasibility of using *E. coli*−produced RBD for the current study is due to the fact that the RBD contains only one glycosylation site Asn−343 that is remote from the RBD−ACE2 interface, and thus its role in ACE2 binding can be excluded.

A Q−TOF mass−spectrometric method was used to determine the molecular weight of RBD before and after purification by SEC with HPLC−Q−TOF−MS (Agilent Technologies, Chandler, AZ, USA). The column used was 300SB−C8 (2.1 mm × 50 mm, 3.5 μm). The mobile phase A was 0.1% formic acid (FA) in water, and B was 0.1% FA in acetonitrile (ACN). The flow rate was 0.2 mL/min. The gradient used was as follows: 0–1 min, 60% A + 40% B; 1–14 min, 100% B; 14–18 min, 100% B; 18–18.1 min, 60% A + 40% B; 18.1–22 min, 60% A + 40% B. Optimal high resolution mass spectrometry (HRMS) parameters were as follows: the electrospray voltage was 5.5 kV; the ion source temperature was 550 °C; ion source gas 1 and gas 2 were both 55 psi; the curtain gas was 35 psi. The data were analyzed with LipidView^TM^ and PeakView^TM^ softwares (AB SCIEX, Framingham, MA, USA).

A RP−HPLC procedure was used to confirm purify and conformational homogeneity of recombinant RBD monitored at absorbance of 225 nm. The Agilent Zorbax 300SB−C18 (4.6 mm × 150 mm, 5 μm) was equilibrated with 0.05% trifluoroacetic acid (TFA) in water (v/v) and peptide components were eluted from the column with a linear gradient from 0 to 60% ACN in 0.05% TFA within 40 min, with a flow rate of 1 mL/min.

### 2.6. Circular Dichroism (CD) Spectroscopy Analysis

For CD analysis, peptide samples were dissolved in 5 mM phosphate buffer (pH 7.0) and RBD was in 50 mM sodium phosphate buffer (pH 7.0) containing 150 mM NaCl, with a concentration of 0.1–0.2 mg/mL. CD spectra were measured on the Chirascan Plus spectropolarimeter v.4.4.0 (Applied Photophysics Ltd., Leatherhead, Surrey, UK) by using a quartz cell of 1.0 mm thickness. The wavelengths used ranged from 190 to 260 nm. Data were collected at 1 nm intervals with a scan rate of 60 nm/min and expressed as delta epsilon (cm^−1^M^−1^). Delta epsilon was calculated as [θ × (MRW × 0.1)/(C × L)/3298], where θ is the ellipticity (in millidegrees), C is the concentration (in mg/mL), L is the path length (in cm), and MRW is the mean residue weight (in Da).

### 2.7. Determination of Peptide−RBD Binding Affinity Using MST

Purified RBD was labeled with the fluorescent dye NT−495−NHS using Monolith NT™ Protein Labeling Kit BLUE−NHS (Cat Nr: L003) according to the manufacturer’s instructions. In this labeling experiment, the fluorescent dye N−hydroxysuccinimide (NHS) esters react with primary amines of lysine side chains and thus covalently crosslink to RBD. The labeled RBD was purified by the gravity flow columns B provided by the kit to remove excess dye. To measure equilibrium binding events by MST and determine the binding affinity (K_D_) of micasin or one of its mutants to RBD, we performed titration experiments with a constant concentration of fluorescent RBD (0.25 μM) against a series of non−fluorescent peptides varying from 0.002–62.5 μM with a two−fold dilution. The buffer used was 5 mM sodium phosphate buffer containing 0.05% Tween 20 (pH 7.0). To achieve this, the samples were loaded into MO NT.115 standard glass capillaries (Cat. No: MO−K022) following a short incubation time and measured using the Monolith NT.115 equipped with a blue filter set (excitation 460–480 nm, emission 515–530 nm) (NanoTemper Technologies, Munich, Bavaria, Germany). In all assays, the initial fluorescence intensity was constant throughout the titration series. K_D_ was calculated using MO. Affinity Analysis program. A representative MST experiment is presented in Figure S2.

## 3. Results

### 3.1. Computational Complex Revealing Extensive Shape Complementarity between Micasin and RBD

**Figure 2:**
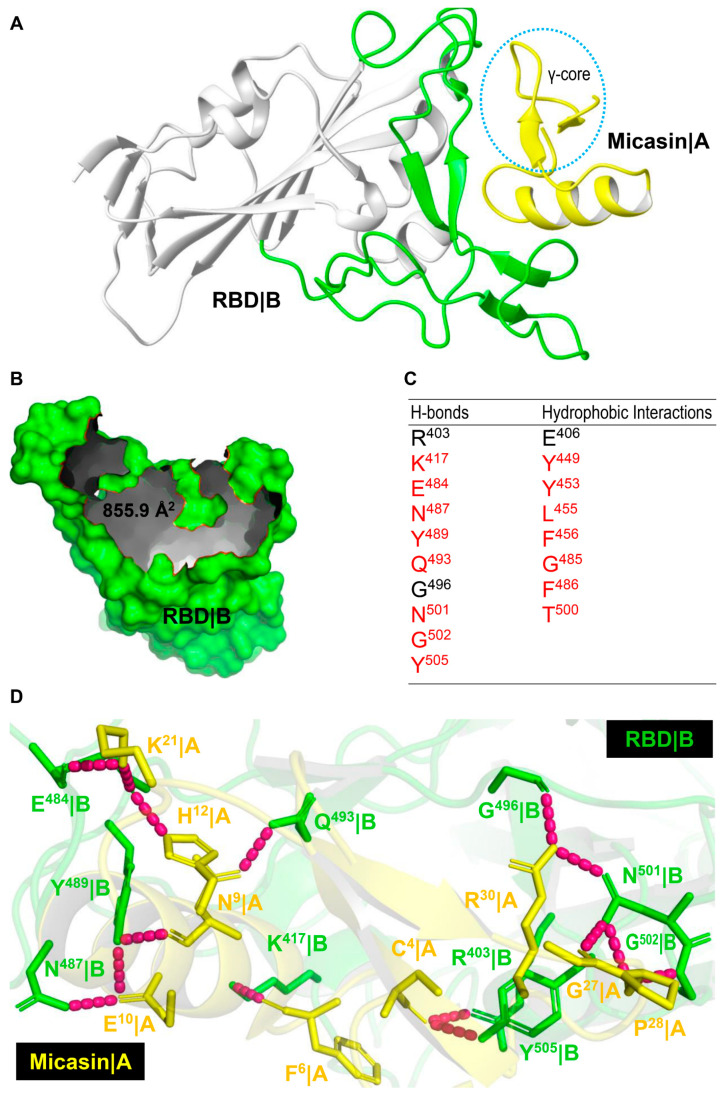
Micasin and RBD interactions. (**A**) Micasin is docked to the crevice of the RBD receptor−binding motif (RBM) using HEX. Micasin|A represents chain A of the complex and shown in yellow with the γ−core circled; RBD|B represents chain B with RBM engaged in interactions with ACE2 shown in green. (**B**) The molecular surface of RBD showing the interface occupied by micasin, with the edge colored in red. (**C**) Residues on RBD making contacts with micasin through hydrogen bonding and hydrophobic interactions are listed here and those also contributing to interactions with ACE2 are colored in red. (**D**) A hydrogen bond network on the micasin and RBD interface. The complex structure is shown as cartoons in a transparency mode in which involved residues are shown as sticks and colored in yellow for micasin and green for RBD; hydrogen bonds are shown as dashed lines in pink.

Micasin is a typical CSαβ−type defensin of 38 residues with a structure that includes two distinct subdomains, namely, the N−terminal helix−associated region (HAR) comprising the α−helix and the N−terminal loop and the C−terminal γ−core region essentially comprising two antiparallel β−strands connected by a loop. These two subdomains are cross−linked by three disulfide bridges (Figure 1A,B), which confer micasin high stability, as revealed by heating at 50 °C for 4 days, not altering its CD spectra (Figure 1C). Using the Hex protein docking program, we generated a series of predicted complexes, from which we chose the first docking pose as the most favorable one for subsequent analysis (Figure 2A). This choice was made because in this complex micasin and RBD form an extensive shape complementarity interface with numerous hydrophobic and hydrogen−bonding interactions. Even more remarkably, as a small peptide of 4−kD, micasin occupies a comparable interface area on RBD (Figure 2B) with ACE2 (855.9 Å^2^ vs. 878.7 Å^2^). Analysis of the complex with LigPlot+ revealed a great number of interacting residues involved in molecular recognition (Figure 1A and Figure 2C). In particular, nearly 50% micasin residues are located on the interface (i.e., Gly−1, Pro−5, Glu−8, Ala−13, Leu−16, Ser−17, Phe−22, Phe−24, and Leu−29 for hydrophobic interactions; and Cys−4, Phe−6, Asn−9, Glu−10, His−12, Lys−21, Gly−27, Pro−28, Arg−30 for hydrogen−bonding interactions) (Figure 1A and Figure 2D). For RBD, 18 residues are located on the interface where 15 are known also to contact ACE2 and three are specific for micasin binding (Figure 2C), indicating that this peptide overall occupies the ACE2 position on RBD.

### 3.2. Micasin Binds RBD with Micromolar Affinity

**Figure 3:**
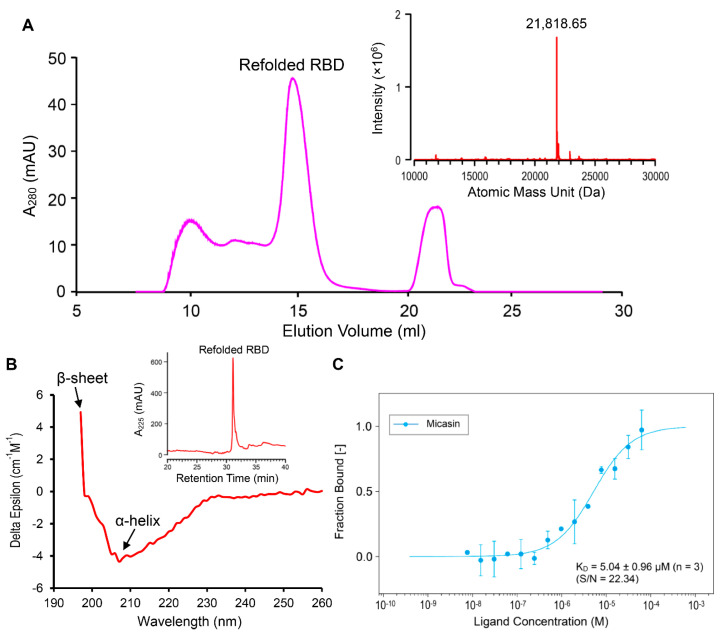
Purification and identification of recombinant RBD and quantification of its binding affinity to NT−495−labelled RBD by microscale thermophoresis (MST). (**A**) Purification of the refolded RBD by SEC. Inset: HPLC−Q−TOF−MS determining the molecular mass. (**B**) CD spectra. Spectral absorbance signatures for β−sheet (a positive maximum at 197 nm) and α−helix (a negative minimum at ~208 nm) are denoted by arrows. Inset: RP−HPLC analysis of the RBD collected from SEC for the detection of its conformational homogeneity. (**C**) The dose−response curve for interactions. Data points derived from the MST traces on the hot region of 20 s indicate the bound fraction generated from the normalized fluorescence (‰) based on fluorescent RBD or the fluorescent RBD and peptide complex against the ligand concentration, from which a dose−response curve was fitted with the K_D_ model for calculation of the binding affinity. Error bars are standard deviations (SDs) from three independent measurements. The experiments were performed at 25 °C at high MST and 20% LED power. S/N, signal−to−noise ratio.

Based on the computational prediction, we experimentally assessed the binding affinity of micasin to RBD using a recombinant native−like RBD protein expressed in a prokaryotic expression system, which contains only one extra N−terminal alanine. Using a modified renaturation method [32], we obtained soluble RBD protein from the *E. coli* inclusion bodies (Figure S1). The product was further purified by size−exclusion chromatography (SEC) (Figure 3A) and the collected peak showed a single component of 21,818.65 Da determined by HPLC−Q−TOF−MS (Figure 3A, inset), precisely matching the theoretical MW of RBD (21,817.49 Da) calculated from the sequence with eight hydrogens removed due to the presence of four disulfide bridges. Analytical RP−HPLC showed that the recombinant RBD adopted a homogeneous conformation (Figure 3B, inset) with a native−like mixed α−helix and β−sheet structure, as identified by its CD spectra (Figure 3B) where a positive maximum at 197 nm signifies β−sheet and a negative minimum around 208 nm signifies α−helix. To determine its affinity to micasin, we titrated a fluorescently labeled RBD with different concentrations of chemically synthesized micasin to record the change of MST traces. The result showed that micasin bound to RBD at a low micromolar concentration with a K_D_ of 5.04 ± 0.96 μM (*n* = 3) (Figure 3C), thus experimentally supporting our computational prediction and indicating that micasin is a novel binder of RBD.

### 3.3. The Role of the γ−Core of Micasin in RBD Binding

**Figure 4:**
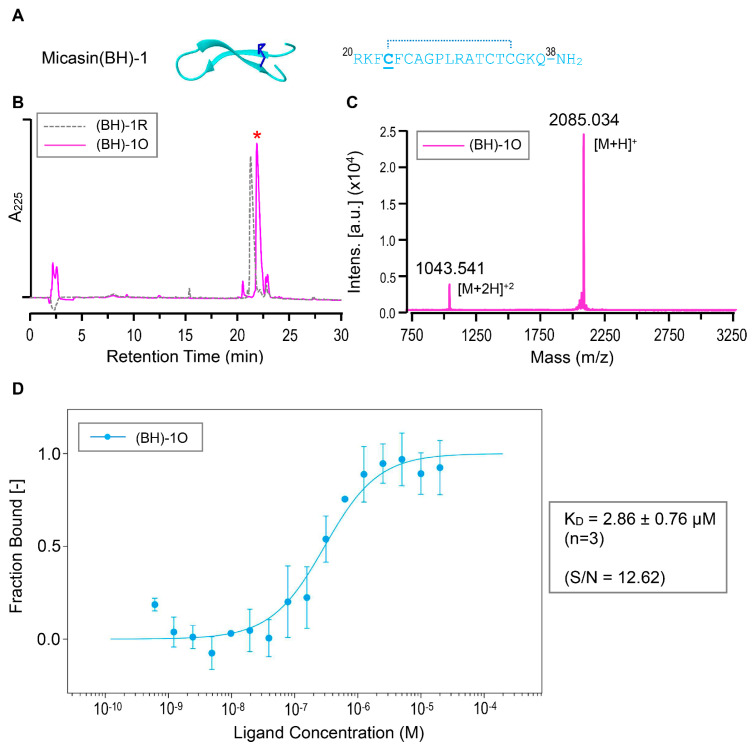
Interactions between (BH)−1 and RBD. (**A**) Sequence and the computational model. The cysteine mutated from the Gly located on β1 is underlined once and the proposed disulfide bridge is denoted by a dotted line. (**B**) RP−HPLC profile of the reduced [(BH)−1R] and oxidized [(BH)−1O] products. The latter marked by a red asterisk was produced via air oxidization. (**C**) MALDI−TOF determining the molecular mass of (BH)−1O showing two peaks corresponding to its singly and doubly protonated forms. (**D**) Quantification of binding affinity of (BH)−1O to NT−495−labelled RBD by MST. The dose−response curve for interactions was fitted with the K_D_ model. Error bars are SDs from three independent measurements. The experiments were performed at 25 °C at high MST and 20% LED power. S/N, signal−to−noise ratio.

Because the γ−core is an independent structural and functional unit of the CSαβ−type defensins [33] and, importantly, it might contain multiple residues involved in the interaction of micasin with RBD (Figure 1), we tested its functional significance in RBD binding. To this end, we chemically synthesized a derivative peptide of micasin corresponding to its γ−core comprising the two β−strands linked by a loop (residues 20−38), in which one Gly to Cys mutation at β1 was introduced to provide stabilization to the isolation peptide, as observed previously [34] (Figure 4A). This synthetic peptide was termed Micasin(BH) −1 (abbreviated as (BH) −1) and its oxidative folding and identification data are presented in Figure 4B,C. MST experiments showed that (BH)−1 had a slightly higher binding affinity than micasin with a K_D_ of 2.86 ± 0.76 μM (Figure 4D). This finding provides support for a role of the γ−core in RBD binding and further strengthens the rationality of the computational complex.

### 3.4. Structure−Based Design Improving the RBD Binding Affinity of Micasin

**Figure 5:**
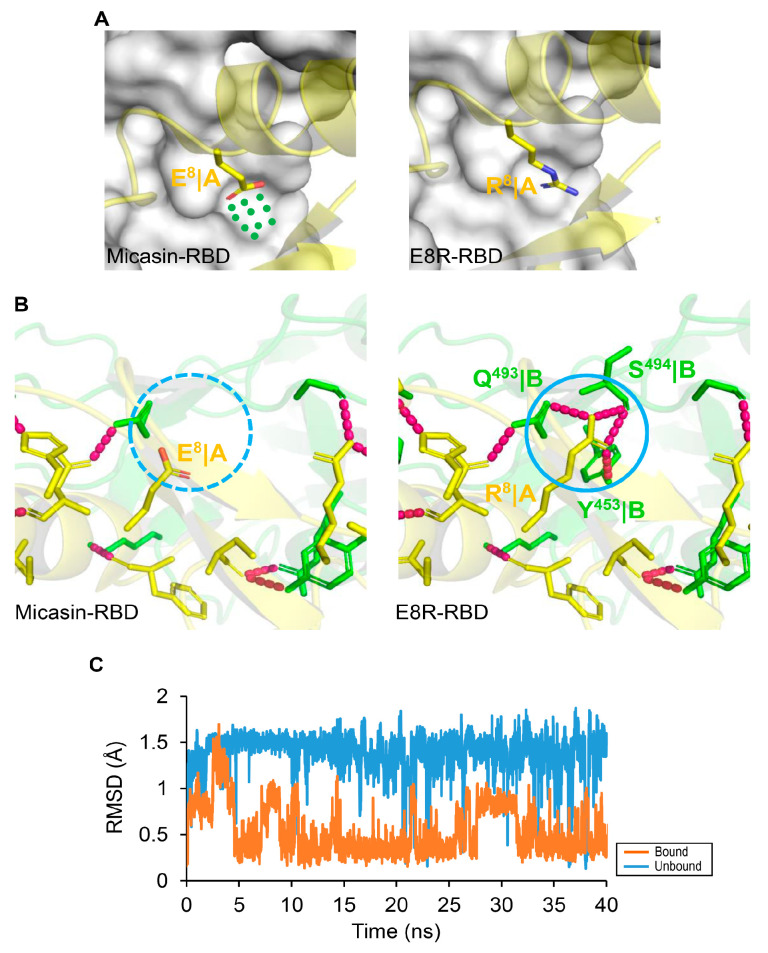
Structure−based design of micasin mutants by targeting the unoccupied surface on RBD. (**A**) The micasin−RBD complex with Glu−8 side−chain shown (**left**). The underutilized cavity by Glu−8 is represented by dots in green. The E8R−RBD complex with Arg−8 side−chain shown (**right**). In these two complexes, peptides are displayed as cartoon model and RBD as molecular surface model. (**B**) Comparison of the two complexes highlighting the side−chain extension from Glu to Arg leading to four new hydrogen bonds between E8R and RBD. (**C**) Side−chain dynamics of Arg−8. The RMSD of Arg−8 extracted from a 40−ns MD simulations with respect to the bound and unbound conformation along the MD trajectory. The RMSD is in reference to the original minimized structure.

To improve the affinity of micasin, we conducted a new design based on analysis of the complex interface to identify unoccupied (or underutilized) surface pockets/cavities on RBD. This strategy has been successfully used in the design of inhibitors targeting protein−protein interactions (PPIs) [35]. We found an underutilized surface cavity on RBD that is spatially close to Glu−8 of micasin and comprises residues Gln−493, Ser−494 and Tyr−453 (Figure 5A). When mutated to Arg−8, the cavity is reasonably well occupied (Figure 5A), consequently leading to a larger interface with an area of 970.5 Å^2^ and four additional hydrogen bonds made between Arg−8 and the cavity residues (Figure 5B), which likely enables a strong interaction in view of the importance of hydrogen bonds in PPIs [36]. MD simulations showed that the binding to RBD reduced the flexibility of Arg−8 compared with the unbound state, as revealed by the difference in their RMSDs (Figure 5C), suggesting that the binding provides a stabilization force for this mutated residue to interact with the cavity.

**Figure 6:**
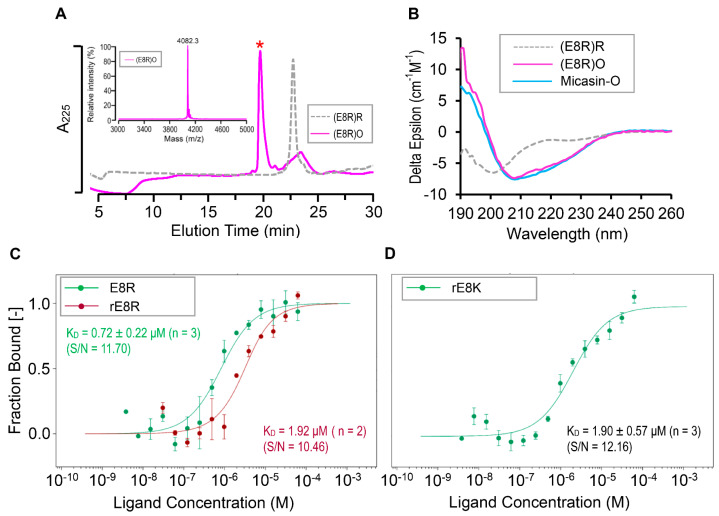
Interactions between micasin mutants and RBD. (**A**) Oxidative folding from the chemically synthesized reduced E8R. The oxidized product is marked by a red asterisk. Inset: the MALDI−TOF image. (**B**) Comparison of CD spectra of E8R between its oxidized (O) and reduced (R) forms. Micasin is used as a control. (**C**) Comparison of E8R binding to RBD between synthetic and recombinant versions. (**D**) Binding of E8K in its recombinant version. Error bars are SDs from two to three independent measurements. In panels (**C**,**D**), the curves were fitted with the K_D_ model. The experiments were performed at 25 °C at high MST and 20% LED power. S/N, signal−to−noise ratio. Notice: preparation of chemically synthesized oxidized micasin and recombinant E8R and E8K samples have been reported previously [24,37].

Subsequently, we prepared a chemically synthesized E8R peptide (Figure 6A) to evaluate its binding affinity to RBD. CD spectral analysis showed that the synthetic peptide adopted a random coli in its reduced form but its oxidized product had similar spectra to those of the unmodified peptide (Figure 6B), indicating that the mutation did not change the structure of micasin. As may be expected, this mutant exhibited a six−fold higher binding affinity to RBD with a K_D_ of 0.72 ± 0.22 μM (*n* = 3) determined by MST (Figure 6C). In parallel, we evaluated the binding affinity of rE8R, a recombinant version of E8R, which had a K_D_ of 1.92 μM *(n* = 2) (Figure 6C). The conservative replacement from Arg to Lys at this site had a similar affinity (1.90 ± 0.57 μM) (*n* = 3) to that of rE8R when a recombinant E8K peptide was used (Figure 6D). However, the introduction of a bulky side−chain tryptophan at this site disrupted the binding, as revealed by our MST experiments (Figure S3), which is most likely due to steric hindrance. Taken together, our data suggest that a cationic residue with a suitable side−chain size at position 8 is key to the improvement of the affinity of micasin.

## 4. Discussion

This study reports for the first time a fungus−sourced defensin with a moderate affinity to the SARS−CoV−2 Spike protein RBD. Using a structure−based approach, we have improved the affinity of micasin and found that micasin and its derivatives share a similar interacting surface on RBD to ACE2 through shape complementarity to establish a hydrogen bond network and hydrophobic interactions, making them a promising template for the design of RBD−targeted inhibitor drugs to control viral entry in the future.

Because the CSαβ−type defensins are rich in various fungi, including Ascomycota, Zygomycota, Basidiomycota and Glomeromycota [38], and especially several of them have shown therapeutic potential [24,39], our study that highlights them as an emerging resource in the design of antiviral drugs would be of important applied value. It is worth mentioning that a moderate binding affinity cannot guarantee that these peptides immediately become a therapeutic agent because they could not compete with human natural ACE2 that has a higher affinity to RBD [29]. To improve their affinity, some current protein engineering technologies may be useful, which include experimental and computational deep mutational scanning, structure−based mutations, ancestral sequence reconstruction and the consensus concept [40,41,42,43,44,45]. In addition, since ACE2 exhibits a higher affinity to RBD when it exists as a dimer, we speculate that the affinity of these defensins would also be improved by linking them to some dimerization scaffolds, such as the immunoglobulin Fc fragments, the ACE2 neck domain, or even some smaller dimerization peptides (e.g., leucine zipper or HLXc) [40,46]. Given the recent emergence of nanomedicine [47,48], multivalent binding of multiple engineered defensins to the surfaces of nanoparticles may be an attractive approach in exploring these peptides into clinical application. In this work, we found that compared with the full−length micasin, the truncated (BH)−1 had a slightly higher affinity to the RBD in the absence of the interacting residue−rich HAR region (Figure 1A). This is, perhaps, not unexpected since a similar case is also observed in other defensins against bacteria [33,34]. In these cases, the smaller peptides might adopt a different mode of action to bind their targets in a more efficient manner.

In addition to the crucial role in the viral entry through interacting with the host receptor [29], the SARS−CoV−2 Spike protein plays multiple roles in pathogenesis of COVID−19. As proof, it works as an inflammation inducer via Toll-like receptor 2−dependent activation of the NF−kB pathway to induce a series of inflammatory factors such as cytokines and chemokines [49]. Its subunit 1 (S1) was found to induce COVID−19−like acute lung injury in transgenic mice and barrier dysfunction in human cells [50]. S1 exhibited a non−receptor−mediated lipid membrane permeabilization and consequently caused the toxicity on human lung cells [51]. Because the membrane−disruptive activity in many cases lacks a cellular specificity, it is likely that such permeabilization would also occur in erythrocytes. If it is true, this finding might well explain the recently observed physical phenotype alterations in blood cells of COVID−19 patients [52]. Therefore, further screening and engineering of fungus−derived high−affinity binders to block the Spike protein could be of value in exploiting drugs to relieve the Spike protein−evoked symptoms of COVID−19.

## 5. Conclusions

This work demonstrates that as a class of naturally occurring, highly thermal stable, and non−toxic peptide scaffolds, fungal defensins show some promise for designing therapeutic drugs to combat COVID−19 by viral neutralization to block infection and binding to the released, free Spike proteins to relieve the patient’s symptoms. Their location on the crevice of the RBD receptor−binding motif (RBM) hits their potential in competing with human ACE2. In particular, the finding that a single−point mutation can improve the binding affinity of micasin to RBD provides a clue for the future direct of engineering. Finally, this work highlights the importance of computer−guided experimental assays in rapidly identifying candidates of RBD binders.

## Data Availability

All relevant data are within the manuscript.

## Supplementary Materials

The following are available online at https://www.mdpi.com/article/10.3390/jof7070553/s1, Figure S1: Characterization of soluble RBD renatured from *E. coli* inclusion bodies; Figure S2: Representative data collected during MST assay of protein−protein interactions; Figure S3: Oxidative folding, characterization and RBD binding affinity of E8W.

## Abbreviations

ACE2, angiotensin converting enzyme 2; ACN, acetonitrile; AMP, antimicrobial peptide; CHCA, α−cyano−4−hydroxycinnamic acid; COVID−19, Coronavirus Disease 2019; CSαβ, cysteine−stabilized α−helical and β−sheet; FA, formic acid; FFT, fast Fourier transform; HAR, helix−associated region; hBD−2, β−defensin 2; HD−5, α−defensin 5; IB, inclusion body; LINCS, linear constraint solver; MALDI−TOF, matrix−assisted laser desorption/ionization time of flight mass spectrometry; MD, molecular dynamics; M^pro^, main protease; MRW, mean residue weight; MST, microscale thermophoresis; NHS, N−hydroxysuccinimide; NPT, number of particles, pressure, and temperature; NVT, number of particles, volume, and temperature; OPLS, optimized potential for liquid simulations; PME, particle mesh Ewald; PPI, protein−protein interaction; RBD, receptor−binding domain; RBM, receptor−binding motif; RMSD, root mean squared deviation; RP−HPLC, reverse-phase high performance liquid chromatography; SARS−CoV−2, Severe Acute Respiratory Syndrome Coronavirus 2; SD, standard deviation; SDS−PAGE, sodium dodecyl sulfate–polyacrylamide gel electrophoresis; SEC, size−exclusion chromatography; TFA, trifluoroacetic acid.

## References

[1] Gallagher T. (2021). COVID19 therapeutics: Expanding the antiviral arsenal. EBioMedicine.

[2] Dai W., Zhang B., Jiang X.M., Su H., Li J., Zhao Y., Xie X., Jin Z., Peng J., Liu F. (2020). Structure-based design of antiviral drug candidates targeting the SARS-CoV-2 main protease. Science.

[3] Ullrich S., Nitsche C. (2020). The SARS-CoV-2 main protease as drug target. Bioorg. Med. Chem. Lett..

[4] Ke Z., Oton J., Qu K., Cortese M., Zila V., McKeane L., Nakane T., Zivanov J., Neufeldt C.J., Cerikan B. (2020). Structures and distributions of SARS-CoV-2 Spike proteins on intact virions. Nature.

[5] DeKosky B.J. (2020). A molecular trap against COVID-19. Science.

[6] Yavari B., Mahjub R., Saidijam M., Raigani M., Soleimani M. (2018). The potential use of peptides in cancer treatment. Curr. Protein Pept. Sci..

[7] Craik D.J., Fairlie D.P., Liras S., Price D. (2013). The future of peptide-based drugs. Chem. Biol. Drug Des..

[8] Curreli F., Victor S.M.B., Ahmed S., Drelich A., Tong X., Tseng C.K., Hillyer C.D., Debnath A.K. (2020). Stapled peptides based on human angiotensin-converting enzyme 2 (ACE2) potently inhibit SARS-CoV-2 infection in vitro. mBio.

[9] Cao L., Goreshnik I., Coventry B., Case J.B., Miller L., Kozodoy L., Chen R.E., Carter L., Walls A.C., Park Y.J. (2020). *De novo* design of picomolar SARS-CoV-2 miniprotein inhibitors. Science.

[10] Maiti B.K. (2020). Potential role of peptide-based antiviral therapy against SARS-CoV-2 infection. ACS Pharmacol. Transl. Sci..

[11] Kurpe S.R., Grishin S.Y., Surin A.K., Panfilov A.V., Slizen M.V., Chowdhury S.D., Galzitskaya O.V. (2020). Antimicrobial and amyloidogenic activity of peptides. Can antimicrobial peptides be used against SARS-CoV-2?. Int. J. Mol. Sci..

[12] Liscano Y., Oñate-Garzón J., Ocampo-Ibáñez I.D. (2020). In silico discovery of antimicrobial peptides as an alternative to control SARS-CoV-2. Molecules.

[13] Bakovic A., Risner K., Bhalla N., Alem F., Chang T.L., Weston W.K., Harness J.A., Narayanan A. (2021). Brilacidin demonstrates inhibition of SARS-CoV-2 in cell culture. Viruses.

[14] Mousavi Maleki M.S., Restamian M., Madanchi H. (2021). Antimicrobial peptides and other peptide-like therapeutics as promising candidates to combat SARS-CoV-2. Expert Rev. Anti Infect Ther..

[15] Lazzaro B.P., Zasloff M., Rolff J. (2020). Antimicrobial peptides: Application informed by evolution. Science.

[16] Yang D., Biragyn A., Hoover D.M., Lubkowski J., Oppenheim J.J. (2004). Multiple roles of antimicrobial defensins, cathelicidins, and eosinophil-derived neurotoxin in host defense. Annu. Rev. Immunol..

[17] Zhang L., Ghosh S.K., Basavarajappa S.C., Muller-Greven J., Penfield J., Brewer A., Ramakrishnan P., Buck M., Weinberg A. (2021). Molecular dynamics simulations and functional studies reveal that hBD-2 binds SARS-CoV-2 Spike RBD and blocks viral entry into ACE2 expressing cells. bioRxiv.

[18] Niv Y. (2020). Defensin 5 for prevention of SARS-CoV-2 invasion and COVID-19 disease. Med. Hypotheses.

[19] Wang C., Wang S., Li D., Wei D.Q., Zhao J., Wang J. (2020). Human intestinal defensin 5 inhibits SARS-CoV-2 invasion by cloaking ACE2. Gastroenterology.

[20] Wang C., Wang S., Li D., Chen P., Han S., Zhao G., Chen Y., Zhao J., Xiong J., Qiu J. (2021). Human cathelicidin inhibits SARS-CoV-2 infection: Killing two birds with one stone. ACS Infect. Dis..

[21] Zhao H., To K.K.W., Sze K.H., Yung T.T., Bian M., Lam H., Yeung M.L., Li C., Chu H., Yuen K.Y. (2020). A broad-spectrum virus- and host-targeting peptide against respiratory viruses including influenza virus and SARS-CoV-2. Nat. Commun..

[22] Zhu S., Gao B., Tytgat J. (2005). Phylogenetic distribution, functional epitopes and evolution of the CSαβ superfamily. Cell. Mol. Life Sci..

[23] Zhu S. (2008). Discovery of six families of fungal defensin-like peptides provides insights into origin and evolution of the CSαβ defensins. Mol. Immunol..

[24] Zhu S., Gao B., Harvey P.J., Craik D.J. (2012). Dermatopytic defensin with antiinfective potential. Proc. Natl. Acad. Sci. USA.

[25] Shafee T.M., Lay F.T., Hulett M.D., Anderson M.A. (2016). The defensins consist of two independent, convergent protein superfamilies. Mol. Biol. Evol..

[26] Vriens K., Cammue B.P., Thevissen K. (2014). Antifungal plant defensins: Mechanisms of action and production. Molecules.

[27] Oppedijk S.F., Martin N.I., Breukink E. (2016). Hit em where it hurts: The growing and structurally diverse family of peptides that target lipid-II. Biochim. Biophys. Acta.

[28] Ritchie D.W. (2003). Evaluation of protein docking predictions using Hex 3.1 in CAPRI rounds 1 and 2. Proteins.

[29] Wang Q., Zhang Y., Wu L., Niu S., Song C., Zhang Z., Lu G., Qiao C., Hu Y., Yuen K.Y. (2020). Structural and functional basis of SARS-CoV-2 entry by using human ACE2. Cell.

[30] Laskowski R.A., Swindells M.B. (2011). LigPlot+: Multiple ligand-protein interaction diagrams for drug discovery. J. Chem. Inf. Model..

[31] Koradi R., Billeter M., Wüthrich K. (1996). MOLMOL: A program for display and analysis of macromolecular structures. J. Mol. Graph..

[32] Zhu S., Gao B., Peigneur S., Tytgat J. (2020). How a scorpion toxin selectively captures a prey sodium channel: The molecular and evolutionary basis uncovered. Mol. Biol. Evol..

[33] Gao B., Zhu S. (2010). Identification and characterization of the parasitic wasp *Nasonia* defensins: Positive selection targeting the functional region?. Dev. Comp. Immunol..

[34] Gao B., Zhu S. (2014). An insect defensin-derived β-hairpin peptide with enhanced antibacterial activity. ACS Chem. Biol..

[35] Rooklin D., Modell A.E., Li H., Berdan V., Arora P.S., Zhang Y. (2017). Targeting unoccupied surfaces on protein-protein interfaces. J. Am. Chem. Soc..

[36] Fersht A.R. (1987). The hydrogen bond in molecular recognition. Trends Biochem. Sci..

[37] Wu J., Gao B., Zhu S. (2016). Single-point mutation-mediated local amphipathic adjustment dramatically enhances antibacterial activity of a fungal defensin. FASEB J..

[38] Wu J., Gao B., Zhu S. (2014). The fungal defensin family enlarged. Pharmaceuticals.

[39] Mygind P.H., Fischer R.L., Schnorr K.M., Hansen M.T., Sönksen C.P., Ludvigsen S., Raventós D., Buskov S., Christensen B., De Maria L. (2005). Plectasin is a peptide antibiotic with therapeutic potential from a saprophytic fungus. Nature.

[40] Chan K.K., Dorosky D., Sharma P., Abbasi S.A., Dye J.M., Kranz D.M., Herbert A.S., Procko E. (2020). Engineering human ACE2 to optimize binding to the Spike protein of SARS coronavirus 2. Science.

[41] Lehmann M., Pasamontes L., Lassen S.F., Wyss M. (2000). The consensus concept for thermostability engineering of proteins. Biochim. Biophys. Acta..

[42] Fowler D.M., Fields S. (2014). Deep mutational scanning: A new style of protein science. Nat. Methods.

[43] Buß O., Rudat J., Ochsenreither K. (2018). FoldX as protein engineering tool: Better than random based approaches?. Comput. Struct. Biotechnol..

[44] Gumulya Y., Gillam E.M. (2017). Exploring the past and the future of protein evolution with ancestral sequence reconstruction: The ‘retro’ approach to protein engineering. Biochem. J..

[45] Glasgow A., Glasgow J., Limonta D., Solomon P., Lui I., Zhang Y., Nix M.A., Rettko N.J., Zha S., Yamin R. (2020). Engineered ACE2 receptor traps potently neutralize SARS-CoV-2. Proc. Natl. Acad. Sci. USA.

[46] Pack P., Plückthun A. (1992). Miniantibodies: Use of amphipathic helices to produce functional, flexibly linked dimeric FV fragments with high avidity in *Escherichia coli*. Biochemistry.

[47] Bobo D., Robinson K.J., Islam J., Thurecht K.J., Corrie S.R. (2016). Nanoparticle-based medicines: A review of fda-approved materials and clinical trials to date. Pharm. Res..

[48] Han Y., Král P. (2020). Computational Design of ACE2-Based Peptide Inhibitors of SARS-CoV-2. ACS Nano.

[49] Khan S., Shafiei M.S., Longoria C., Schoggins J., Savani R.C., Zaki H. (2021). SARS-CoV-2 Spike protein induces inflammation via TLR2-dependent activation of the NF-κB pathway. bioRxiv.

[50] Colunga Biancatelli R., Solopov P., Sharlow E.R., Lazo J.S., Marik P.E., Catravas J.D. (2021). The SARS-CoV-2 Spike Protein Subunit 1 induces COVID-19-like acute lung injury in Κ18-hACE2 transgenic mice and barrier dysfunction in human endothelial cells. Am. J. Physiol Lung Cell Mol. Physiol..

[51] Asandei A., Mereuta L., Schiopu I., Park J., Seo C.H., Park Y., Luchian T. (2020). Non-receptor-mediated lipid membrane permeabilization by the SARS-CoV-2 Spike protein S1 subunit. ACS Appl Mater. Interfaces..

[52] Kubánková M., Hohberger B., Hoffmanns J., Fürst J., Herrmann M., Guck J., Kräter M. (2021). Physical phenotype of blood cells is altered in COVID-19. Biophys. J..

