# Supplementary material for: A Fungal Defensin Targets the SARS−CoV−2 Spike Receptor−Binding Domain
Source: J Fungi (Basel). 2021 Jul 12;7(7):553. doi: 10.3390/jof7070553 (PMC8304516; doi:10.3390/jof7070553)
Supplement: Supplementary file 1 [file jof-07-00553-s001.zip › jof-1297513-supplementary.pdf]

Supplementary Material

Supplement to: Bin Gao, Shunyi Zhu. A Fungal Defensin Targets the SARS-CoV-2 Spike Receptor-Binding Domain

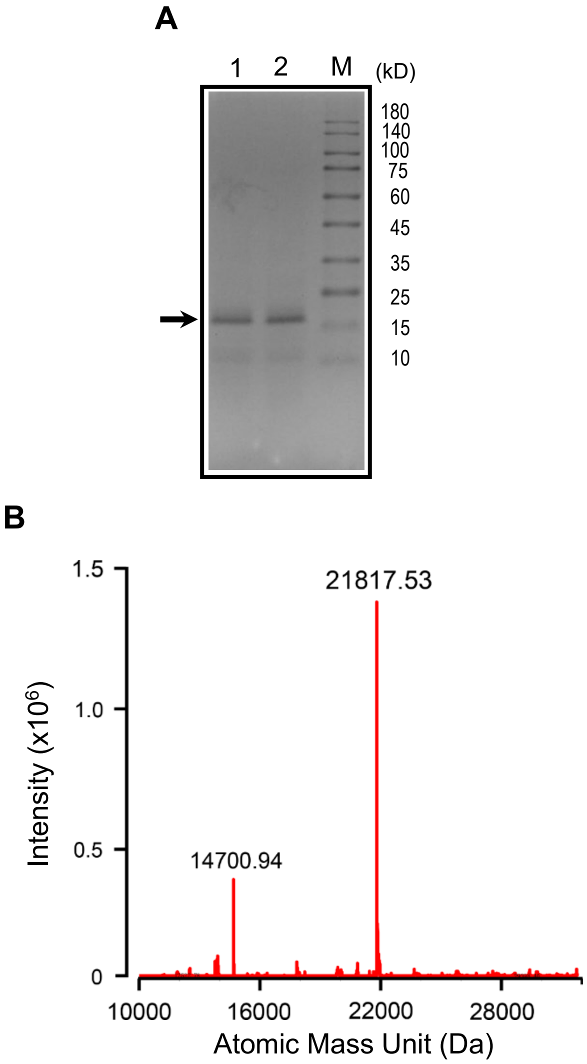

Figure S1. Characterization of soluble RBD renatured from *E. coli* inclusion bodies. (**A**) SDS-PAGE analysis of the renatured RBD obtained through ultra-filtered concentration with a 3 kD MWCO centrifugal device (Millipore, Bedford, MA, USA). The RBD band is marked by an arrow. (**B**) HPLC-Q-TOF-MS detection of the renatured RBD. Notice: 14700.94 Da was an unidentified component in the renaturation product.

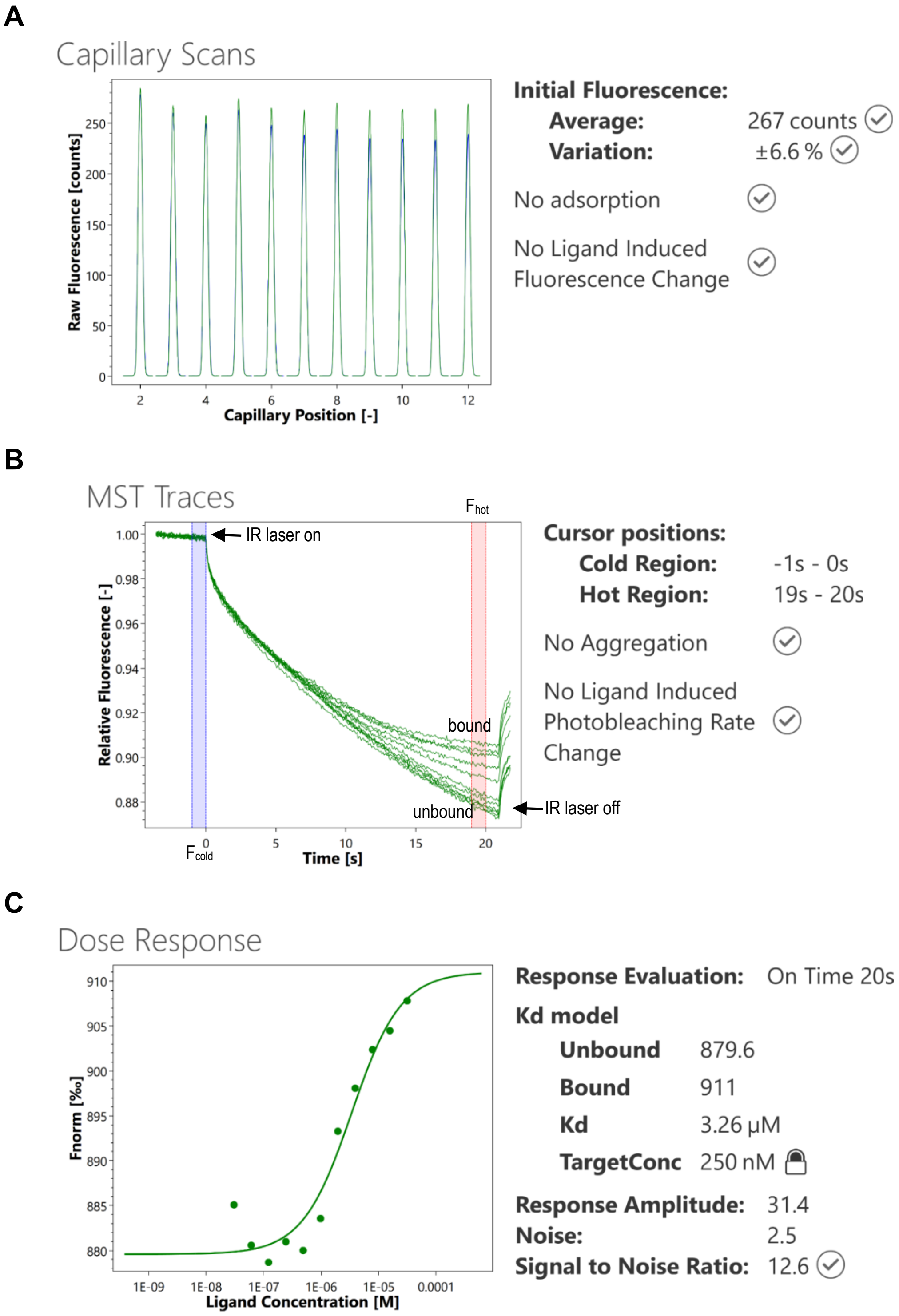

Figure S2. Representative data collected during MST assay of protein-protein interactions. (**A**) Fluorescence capillary scans showing no ligand-induced fluorescence change across a series of capillaries that contained a constant concentration (250 nM) of RBD labelled by the BLUE-NHS fluorophore NT-495 and unlabeled rE8R at variable concentrations. (**B**) Typical MST traces for the peptide showing positive thermophoresis. (**C**) Binding of rE8R to the labelled RBD. The dose-response curve was fitted with a signal to noise ratio of 12.6.

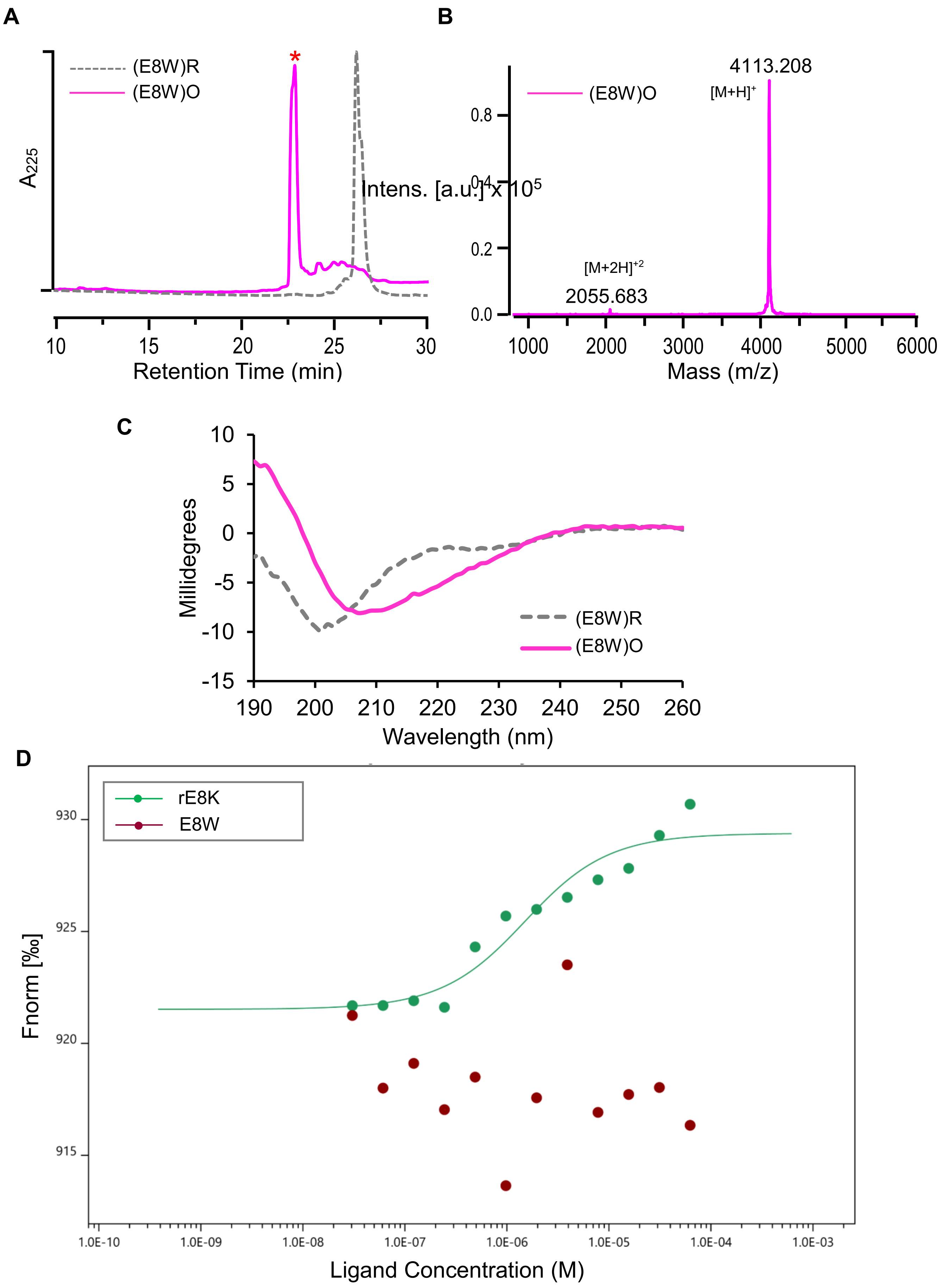
A
(E8W)R
(E8W)O
A225
10
15
20
25
30
Retention Time (min)
B
(E8W)O
4113.208
[M+H]+
[M+2H]+2
2055.683
0.8
0.4
0.2
0.0
Intens. [a.u.] x 10^5
1000
2000
3000
4000
5000
6000
Mass (m/z)
C
10
5
0
-5
-10
-15
Millidegrees
(E8W)R
(E8W)O
190
200
210
220
230
240
250
260
Wavelength (nm)
D
rE8K
E8W
930
925
920
915
Fnorm [‰]
1.0E-10
1.0E-09
1.0E-08
1.0E-07
1.0E-06
1.0E-05
1.0E-04
1.0E-03
Ligand Concentration (M)

Figure S3. Oxidative fold-ing, characterization and RBD binding affinity of E8W. **(A)** RP-HPLC of the reduced and oxidized products. The latter was produced via air oxidization. (**B**) MALDI-TOF determining the molecular weight of the oxidized product showing two peaks corresponding to its singly and doubly protonated forms. (**C**) Comparison of the circular dichroism spectra between the reduced and oxidized products. (**D**) Quantification of binding affinity between RBD and E8W or rE8K by MST. Data points indicate the normalized fluorescence (‰) generated by fluorescent RBD or the fluorescent RBD and peptide complex, from which curves are fitted with the K_D model.
